# Phylogeny, structural patterns, and polymorphisms in *Dyckia* spp. from the Espinhaço mountain range based on complete chloroplast genome

**DOI:** 10.3389/fpls.2025.1549351

**Published:** 2025-06-19

**Authors:** João Victor Da Silva Rabelo-Araujo, Ana Flávia Francisconi, Caroline Bertocco Garcia, Cássio Van Den Berg, Otavio Batista de Castro Ribeiro, Ana Paula da Silva Marques, Matheus Scaketti, Ana Cristina Silva Amoroso Anastacio, Maria Imaculada Zucchi

**Affiliations:** ^1^ Universidade Estadual de Campinas, Instituto de Biologia, Campinas, Brazil; ^2^ Escola Superior de Agricultura Luiz de Queiroz, Universidade de São Paulo, São Paulo, Brazil; ^3^ Department of Biological Sciences, Universidade Estadual de Feira de Santana, Feira de Santana, Bahia, Brazil; ^4^ Instituto Rupestris, Minas Gerais, Brazil; ^5^ Department Long-term Strategic Studies, Vale S.A, Minas Gerais, Brazil

**Keywords:** rocky outcrops, plastomes, biodiversity, bromeliads, molecular markers

## Abstract

*Dyckia* spp. are xeromorphic bromeliads, with diversity centered in the ferruginous rocky outcrops of the Espinhaço mountain Range in Brazilian tropical savana. Due to their recent radiation – during the Pliocene – along with their vast phenotypic plasticity and the limited herbarium records, many species in the genus present challenges for correct identification and the development of robust conservation strategies. We sequenced the complete chloroplast genome of six rare *Dyckia* species in an effort to identify hotspots of diversity that could serve as molecular markers capable of distinguishing species and elucidating phylogenetic relationships within the genus. The plastome sizes of the species ranged from 159,689 bp to 159,264 bp, and the GC content was highly consistent across all species, varying narrowly between 37.2% to 37.3%. Despite the high structural similarity, polymorphism analyses identified three regions of high polymorphic diversity—the *clpP1* and *psa*1 genes, and the intergenic spacer region between *trnT* and the *trnL* — that may serve as molecular markers within the genus. Additionally, we detected a high number of SSRs (637), dispersed repeats (208), SNPs (1035), and indels (823) among the species compared to other bromeliads. Phylogenetic analyses using whole plastid genomes showed low variability among species, associating them with their geographic distribution. These results not only highlight the structural variability of the *Dyckia* plastid genome but also provide new molecular tools for the identification of related species, enhancing the development of conservation protocols for these bromeliads.

## 1 Introduction

*Dyckia* Schult. & Schult.f. are xenomorphic bromeliads with terrestrial or saxicolous habits and CAM photosynthetic metabolism. These plants are morphologically characterized by the lateral emission of a racemose inflorescence, rosette leaves with well-developed spines on the leaf margins, variations in succulence level, extranuptial nectaries, and red, yellow, or orange petals (Smith and Downs, 1974; Guarçoni et al., 2017). Although the genus has approximately 170 described species with wide distribution throughout South America, 80% of them are endemic to Brazil (Leme and Kollmann, 2011). The region with the highest endemism of *Dyckia* species is the southern portion of the Espinhaço mountain range in the Brazilian state of Minas Gerais, an ecosystem characterized by a heterogeneous alpine landscape, full of ferruginous rocky outcrops locally known as “canga” with dry soils and nutrient scarcity (Versieux and Wendt, 2006; Silveira et al., 2016).

According to estimates based on plastid markers, *Dyckia* underwent a recent massive radiation process between 4.6 to 2.9 million years ago, still in the Pliocene (Krapp et al., 2014). The frequent temperature variations during this period, as well as the heterogeneous landscape of the Campos Rupestres, may have contributed to the frequent isolation and fragmentation of populations of xeric plants due to altitude variations throughout this ecosystem, promoting widespread allopatric speciation, as well as recurrent introgression, of several species along the entire Espinhaço mountain range (Antonelli et al., 2009; Antonelli et al., 2010).Despite the significant diversity of *Dyckia* in this region, the low morphological heterogeneity between related species, the scarcity of records in herbaria and collections, as well as the lack of distribution and population diversity data, often lead to errors in endemic species identification, including at the molecular level (Pinangé et al., 2020). In addition to the identification difficulties of *Dyckia* in the region, many rare species still face constant threats from human activities such as mining, agriculture, and livestock farming, as well as recurrent spontaneous fires (Versieux and Wendt, 2007; Leme et al., 2012). This scenario underscores the urgent need for reliable molecular markers capable of distinguishing morphologically similar species and supporting effective conservation strategies.

Phylogenetic relationships in *Dyckia* have so far been based on some universal plastid regions used for Bromeliaceae phylogenies (*matK*, *rps16* intron, *petD* intron, *rpl32-trnL*, *rps16-trnK*, *trnS-ycf3*, and *trnD-trnT*), the use of the nuclear locus *phyC*, AFLP molecular markers and morphological analyses (Knowles and Chan, 2008; Philippe et al., 2011; Krapp et al., 2014). Although previous molecular studies have included up to 106 *Dyckia* species and confirmed the monophyly of the genus, the resulting phylogenies consistently recover clades organized by geographic distribution rather than by taxonomic affinity. These phylogenies often exhibit low resolution among closely related species, frequently resulting in large polytomies and poorly informative trees.

The limited resolution of phylogenetic trees is a recurrent issue in taxonomic groups that have undergone recent speciation, as the brief intervals between evolutionary events does not allow enough time for genetic variation to accumulate between taxa. This issue is particularly pronounced especially when using few markers with low mutation rate, such as plastid sequences with limited variability, which are frequently uninformative for distinguishing closely related species (Knowles and Chan, 2008; Philippe et al., 2011). This is also true for other phylogenetic analyses in taxa within the Bromeliaceae, such as *Puya*, *Tillandsioideae*, and *Hechtioideae*, whose rapid and recent speciation was strongly influenced by climatic and geographic variations in the last 10 million years (Jabaily and Sytsma, 2010; MaChado et al., 2020; Rivera-Martínez et al., 2022).

In contrast, with the advancement of Next-Generation Sequencing (NGS) techniques, new phylogenomic analyses based on whole plastome sequences have become a powerful tool for resolving phylogenies in complex and recent taxa, providing new insights into plastome evolution and aiding in the identification of reliable new diversity hotspots to function as powerful molecular markers (Wang et al., 2021; Liu et al., 2022; Ren et al., 2022).

Land plants chloroplast genomes are highly conserved, ranging in size from 120 to160 kb, and are typically organized into a quadripartite structure comprising large (LSC) and small (SSC) single-copy regions and two inverted repeat (IR) regions (Dobrogojski et al., 2020). Containing between 100–150 functional genes, as well as tRNAs and rRNAs, the plastid genome genes are commonly involved in photosynthesis pathways, but also in fatty acid synthesis, and nucleic acid transcription and translation (Wicke et al., 2011; Olejniczak et al., 2016). Although plastid genes are characterized by a low mutation rate, structural rearrangements, gene losses, and the contraction of repetitive regions can be used to infer evolutionary relationships and serve as effective molecular markers (Cauz-Santos et al., 2020; Vera-Paz et al., 2022; Ramírez-Morillo et al., 2023).

In this study, we provide seven newly sequenced complete chloroplast genomes of *Dyckia* representing six rare species restricted to the Espinhaço mountain range. We aim to 1) identify potential molecular diversity hotspots to serve as new markers capable of discriminating species; 2) To detect repetitive regions, structural variations and possible syntenies with other available Bromeliaceae species; and to assess the effectiveness of phylogenies based on whole plastome sequences in elucidating interspecific relationships among closely related species of the genus.

## 2 Materials and methods

### 2.1 Plant material and DNA extraction

The six species of *Dyckia* analyzed in this study are restricted to rocky outcrops in the southern portion of the Espinhaço Mountain Range, in the state of Minas Gerais, Brazil. Samples of *Dyckia consimilis* (Mez), as well as individuals tentatively referred to as *Dyckia aff*. *trichostachya* and *Dyckia* sp., were collected from remnant populations across the Sinclinal Moeda, a mountainous region located south of Belo Horizonte, in the state of Minas Gerais, Brazil. The latter two could not be identified to the species level due to the absence of inflorescence, a key morphological trait for species delimitation in *Dyckia*. These individuals may represent undescribed taxa.

Due to the pronounced morphological variability observed in *Dyckia consimilis*, even among populations in close geographic proximity—and its occasional misidentification with related taxa such as *D. macedoi* (L.B.Sm.) and *D. schwackeana* (Mez)—two individuals of this species were included in the sampling. This strategy was adopted to better represent the potential intraspecific genetic diversity at the plastid genome level.

Samples of *Dyckia rariflora* (Schult. & Schult.f.) and *Dyckia elata* (Mez) were obtained from populations occurring on rocky outcrops of Serra de Antônio Pereira, in the municipality of Catas Altas, while samples of *Dyckia densiflora* were collected in Serra da Piedade, located in Caeté, Minas Gerais, Brazil. All the species analyzed here are included in the “Rede Propagar”, a plant conservation initiative for threatened species of the Campos Rupestres, developed in partnership between Vale S.A. and the São Paulo Agency for Agribusiness Technology (APTA).

Leaf samples from seven specimens of the species described above were kept in saturated NaCl-CTAB solution in a refrigerator at 8°C until DNA extraction (Rogstad, 1992). Total genomic DNA was isolated using approximately 200mg of leaves per sample. Leaves were macerated using liquid nitrogen, and the extraction was performed using a modified protocol of DNeasy Plant Maxi Kit (Qiagen). Extracted DNA samples were quantified in a 1% agarose gel and stained with GelRed (Sigma-Aldrich) for visualization under ultraviolet light using 1 Kb Plus DNA Ladder a reference. Samples were quantified using the Qubit Broad range kit fluorometer (Sigma-Aldrich).

### 2.2 Chloroplast genome sequencing, assembly, and annotation

The genomic libraries of the seven specimens were constructed using 250 ng of total genomic DNA using the Illumina DNA Prep kit following the manufacturer’s instructions. The libraries were sequenced in Illumina NextSeq 2000 platform in a paired-end sequencing (2 × 100 bp) in the Life Sciences Core Facility (LaCTAD) from the State University of Campinas (UNICAMP). The assembly of Chloroplast DNA from *Dyckia* species was conducted using two different strategies: First, we employed GetOrganelle v 1.7.3.1 (https://github.com/Kinggerm/GetOrganelle/) using as seed, the plastome of *Pitcairnia breedlovei* (NC_080307.1), available in GenBank (https://www.ncbi.nlm.nih.gov/nuccore/NC_080307.1). Despite multiple attempts with GetOrganelle for the chloroplast genome assembly of *D. rariflora*, we were unsuccessful. For this species, we therefore used NovoWrap (https://github.com/wpwupingwp/novowrap), following the parameters described in the online manual for the correct assembly of plastomes (Jin et al., 2020; Wu et al., 2021).To assess the continuity of plastome alignment we use BWA v.07.17 (https://github.com/lh3/bwa) (Abeel et al., 2012) for mapping DNA sequences against a reference chloroplast genome of *Ananas comosus* (NC_026220.1). Subsequently, Samtools v.1.13. (https://github.com/samtools/samtools) was employed to organize the data generated from the alignment by estimating the average coverage, and to visualize the mapping, we used Integrative Genomics Viewer (IGV) (https://igv.org/) and Geneious prime v2023 1.2 (https://www.geneious.com). The plastome annotation was performed with the Chlorobox platform GeSeq (Organellar Genome Annotation) in which parameters were set to identify protein-coding sequences (CDS), rRNAs, and tRNAs by referencing chloroplast sequences and homologies through BLAST and the 3^rd^ Party stand-alone annotator Chloe. After GeSeq annotation, we performed manual correction of start and stop codons, and verification of pseudogene and intron positions using GenomeView (Abeel et al., 2012; Tillich et al., 2017). Finally, chloroplast circular genome maps were generated using OGDRAW (Greiner et al., 2019).

### 2.3 Chloroplast genome structure comparison and detection of nucleotide divergence hotspots, SNPs, and indels

Two multiple progressive sequence alignments in Mauve v.2.4 were used for the comparative plastid analysis in *Dyckia* (Darling et al., 2004). First, using the chloroplast genome of *Pitcairnia atrorubens* (Genbank: NC_085612.1) as reference, we aligned the seven sequenced *Dyckia* specimens here: two individuals of *Dyckia consimilis*, *D. rariflora, D. elata*, *D. densiflora*, *D.* aff. *trichostachya* and *Dyckia* sp. The second analysis aimed to understand the structural synteny of the chloroplast genome in Bromeliaceae. The analysis was conducted using our set of sequenced *Dyckia* species, and seven Bromeliaceae samples representing seven additional subfamilies available in GenBank: Brocchinioideae, Bromelioideae, Hechtioideae, Puyoideae, Tillandsioideae, Navioideae, and Lindmanioideae (**Supplementary Table S1**).

Similarly, to assess potential events of expansions and contractions of the IR regions, the genes present in the borders of LSC and SSC were annotated and manually compared to detect potential polymorphic regions, we performed pairwise alignments between the chloroplast genomes of the second alignment using MAFFT v.7 (Katoh et al., 2002). Employing the DnaSP v.5 program, we conducted a sliding window analysis (with a window length of 200 bp and a step size of 50 bp) to identify nucleotide divergence hotspots (Librado and Rozas, 2009). Additionally, the first alignment among the seven plastomes was used to nail down small insertions/deletions (indels) in the sequences (**Supplementary Table S2**) and to identify single nucleotide polymorphisms (SNPs) among the genomes (**Supplementary Table S3**). All positions of nucleotide divergence hotspots, indels, and SNPs were manually identified using the annotations of the chloroplast genomes obtained from GE-SEQ. Heatmaps were generated from SNP and indel data using the pheatmap package in the R platform v. 4.2.2.

### 2.4 Identification of SSRs and dispersed repeats

To detect simple sequence repeats (SSRs) of 1–6 nucleotides, we used the virtual MISA package (available at https://webblast.ipk-gatersleben.de/misa/). The criteria for identifying SSR motifs (**Supplementary Table S4**) in the chloroplast genome were as follows: SSRs ranging from one to six nucleotides in length, with a minimum repeat number of 10, 5, and 4 units for mono-, di-, and trinucleotide SSRs, respectively, and three units for tetra-, penta-, and hexanucleotide SSRs (Beier et al., 2017). REPuter was used to investigated the presence of four types of dispersed repeats: forward, reverse, palindrome and complement sequences (**Supplementary Table S5**). The criteria for the identification were: minimum repetition size ≥ 30 bp and sequence identity ≥ 90% (Hamming distance = 3). The location of SSRs and dispersed repeat sequences were manually annotated between the species (Kurtz and Schleiermacher, 1999).

### 2.5 Phylogenomic studies

The plastomes of all seven samples sequenced in this study were aligned to 14 Bromeliaceae species from the genbank (**Supplementary Table S1**) using the MAFFT tool (Katoh et al., 2002). *Lindmania* sp. (genbank accession OQ308827) was used as an outgroup based on previous phylogenetic studies in Bromeliaceae (Givnish et al., 2014). Of the 206,120 bp of the aligned matrix, we used positions 45163-211048, which were completely collinear between all samples, with a total of 165,996 characters, including the entire LSC, SSC, IRa, IRb, and all genes, spacers and introns. Phylogenetic analysis and model selection were performed with IQTREE2 (Minh et al., 2020) Model selection was performed with ModelFinder embedded in IQTREE2, and phylogenetic analysis was performed under Maximum Likelihood criterion, and 1,000 ultrafast-bootstrap pseudoreplicates, with the -BNNI option, to avoid overestimation due to model violations along the heterogeneous matrix. The bootstrap consensus tree was imported to FigTree 1.4 (https://github.com/rambaut/figtree/releases) and later edited in Inkscape.

## 3 Results

### 3.1 Organization of the *Dyckia* species chloroplast genomes

**Figure 1:**
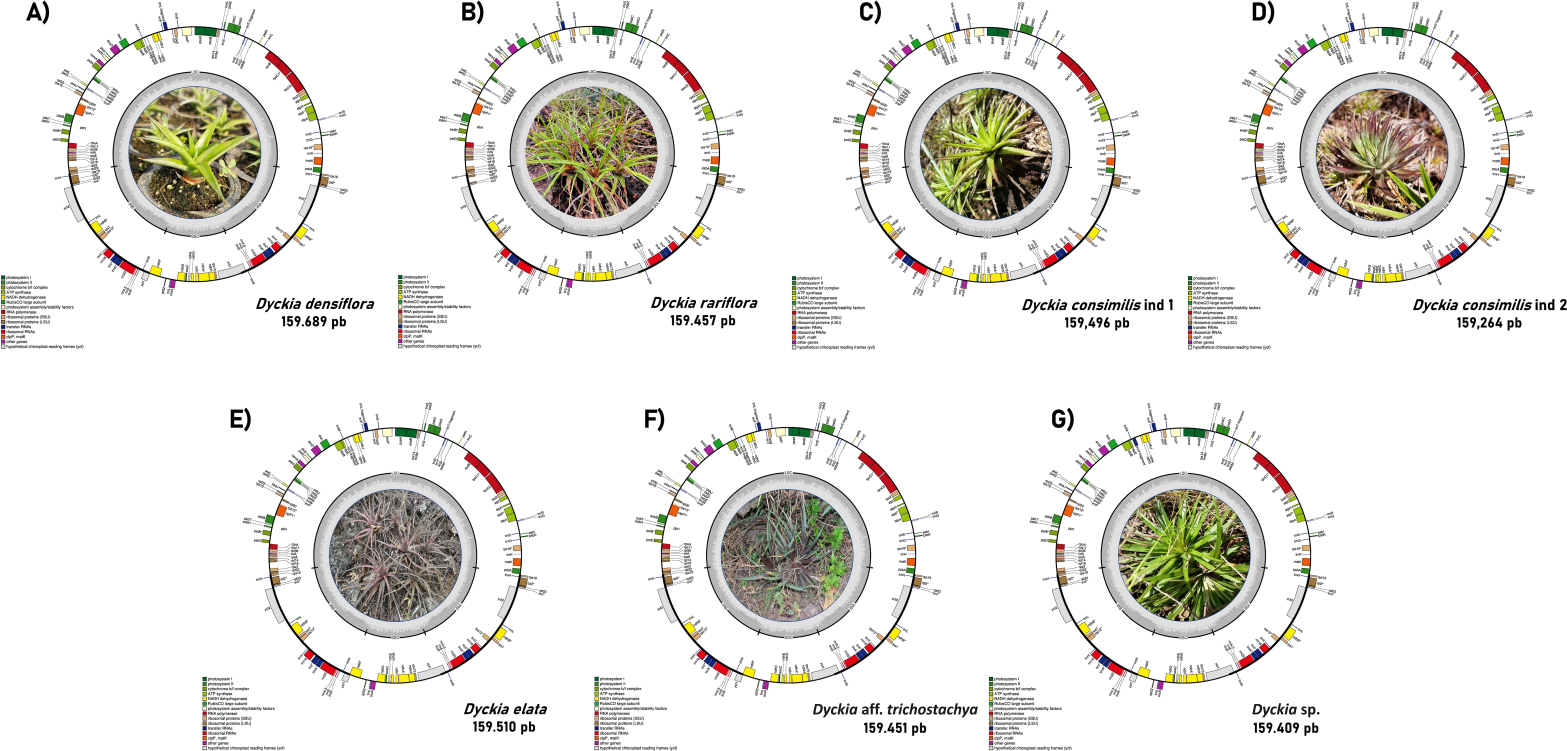
Circular representation of Cloroplastidial genome of *Dyckia* spp. The color within the genes represents their categorical function, while the inner circle in gray represents the GC content along the plastome. Genes shown inside the circle have a clockwise orientation, while those outside have a counterclockwise orientation. **(A)** *Dyckia densiflora*; **(B)** *Dyckia rariflora*; **(C)** *Dyckia consimilis* ind 1; **(D)** *Dyckia consimili*s ind 2; **(E)** *Dyckia elata*; **(F)** *Dyckia* aff. *trichostachya*; **(G)** *Dyckia* sp.

The chloroplast genome of *D. consimilis*, *D. rariflora, D. elata, D. densiflora, D.* aff. *trichostachya, and D.* sp had the typical quadripartite circular structure consisting of one large single copy (LSC), one small single copy (SSC), and two inverted repeats (IRB and IRA) regions (**Figure 1**). The plastome size ranges from 159,689 pb (*Dyckia densiflora*) to 159,264 pb (*Dyckia consimilis* ind 2) with all plastomes displaying comparable lengths for the LSC, SSC, and IR regions. The GC content was highly consistent across all species, ranging between 37.2% and 37.3%. (**Table 1**).

**Table 1:** General features of chloroplast genomes of seven *Dyckia* taxa.

Taxa	*Dyckia consimilis* ind 1	*Dyckia consimilis* ind 2	*Dyckia rariflora*	*Dyckia elata*	*Dyckia* aff. *trichostachya*	*Dyckia densiflora*	*Dyckia* sp.
Total cpDNA size (bp)	159.495	159.264	159.457	159.510	159.451	159.689	159.409
LSC length (bp)	87.331	87.104	87.296	87.348	87.289	87.495	87.229
SSC length (bp)	18.714	18.710	18.711	18.712	18.712	18.734	18.730
IR length (bp)	26.725	26.725	26.725	26.725	26.725	26.725	26.725
Total GC content (%)	37,2	37,3	37,3	37,2	37,3	37,2	37,3
Total number of genes	132	132	132	132	132	132	132
Protein-coding genes	86	86	86	86	86	86	86
rRNA genes	8	8	8	8	8	8	8
tRNA genes	40	40	40	40	40	40	40

LSC, Large single copy; SSC, small single copy; IR, inverted repeat.

*Dyckia* plastomes present the same amount of genes (132), with 86 protein-coding genes, eight ribosomal RNAs (rRNAs), and 40 transfer RNAs (tRNA) (**Figure 1**). Among the protein-coding genes present in *Dyckia* spp. five were associated with Photosystem I, 14 with Photosystem II, six with Cytochrome b/f complex, six genes with ATP synthase, and 11 genes responsible for NADH dehydrogenase (**Table 2**). Regarding the hypothetical chloroplast reading frames (ycf), all *Dyckia* spp. exhibited a small fragment of ycf1 at the border of the IRB and SSC regions. This distinctive characteristic was simultaneously found in all *Dyckia* spp. exclusively through the annotation software Chloe, which was the unique gene exclusive to this third-party stand-alone annotator.

**Table 2:** Chloroplastidial genes in *Dyckia* spp.

Group of genes	Gene name
Photosystem I (5)	*psaA, psaB, psaC^c,^ psaI, psaJ*
Photosystem II (14)	*psbA, psbB, psbC, psbD psbE, psbF, psbH, psbJ, psbK, psbL, psbl, psbM, psbT, psbZ*
Cytochrome b/f complex (6)	*petA, petB* ^a^ *, petD, petG, Petl, petN*
ATP synthase (6)	*atpA, atpB, atpE, atpF, atpH, atpl*
NADH dehydrogenase (11)	*ndhA^c^, ndhB^,d^*, *ndhD^d^, ndhE^d^, ndhF^c^, ndhG^d^, ndhH^d^, ndhJ, ndhK, ndhl^d^, nhdC*
RubisCO large subunit (1)	*rbcL*
Photosystem assembly/stability factor (3)	*pafI^b^, pafII, pbf1*,
RNA polymerase (4)	*rpoA, rpoB, rpoC1, rpoC2*
Ribosomal proteins (SSU) (13)	*rps2, rps3, rps4, rps7 ^d^, rps8, rps11, rps12^a,d^, rps14, rps15 ^c^, rps16, rps18, rps19^d^*
Ribosomal proteins (LSU) (9)	*rpl2^a,d^, rpl14, rpl16, rpl20, rpl22, rpl23^d^, rpl32^c^, rpl33, rpl36*
Transfer RNAs (25)	*trnA^a,d^, trnC, trnD, trnE, trnF, trnfM, trnG, trnG-FRA, trnH, trnI ^a,d^, trnK, trnL, trnL-fragment ^a^, trnM, trnN, trnP, trnQ, trnR, trnS, trnT, trnT-fragment, trnV, trnV-fragment, trnW, trnY*
Ribossomal RNAs (4)	*rrrn 4.5 d, rrrn 5 d, rrrn16 ^d^, rrrn23 ^d^*
ClpP, MatK (2)	*ClpP1^b^, matK*
Hypotetical chloroplast Reading frames (ycf) (2)	*yfc1^c,d^, yfc2^d^*
Other genes (3)	*InfA, cemA, accD*

a Gene containing one intron.

b Gene containing two introns.

c Gene occurring in small single copy region.

d Two gene copies in the inverted repeats.

Additionally, some genes showed slight variations in length among species. Associated with the coding of the ATP synthase F subunit, the gene *atpF* had a length of 1414 bp in all species, except for *Dyckia* sp., whose length was slightly lower at 1398 bp. Similarly, *ndhD* in *Dyckia consimilis* and *Dyckia densiflor*a possess a length of 1506 bp, however, in *D. rariflora*, *D. elata*, *D.* aff. *trichostachya*, and *D*. sp., the gene had a length of 1527 bp. These minor differences, although small and possibly negligible in functional terms, may be attributed to the insertion or deletion of indels within the gene sequences.

The chloroplast genomes revealed 15 unique genes containing introns: 12 in protein-coding genes (*rpl2*, *ndhB*, *rps12*, *ndhA*, *petB*, *clpP1*, *pafI*, *rpoC1*, *atpF*, *rps16*) and three (*trnI*, *trnA*, *trnL*-fragment) within tRNA-coding genes. Five genes were duplicated in the inverted repeats (*rpl2*, *ndhB*, *rps12*, *trnI*, *trnA*). Only two genes (*clpP1* and *pafI*) comprised two introns, while the others contained only a single intron. The largest intron was found in *ndhA* (1061 bp) and the smallest in *petB* (215 bp) in the SSC and the LSC region respectively.

### 3.2 Structural comparison for *Dycki*a spp. plastomes with related species in Bromeliaceae

**Figure 2:**
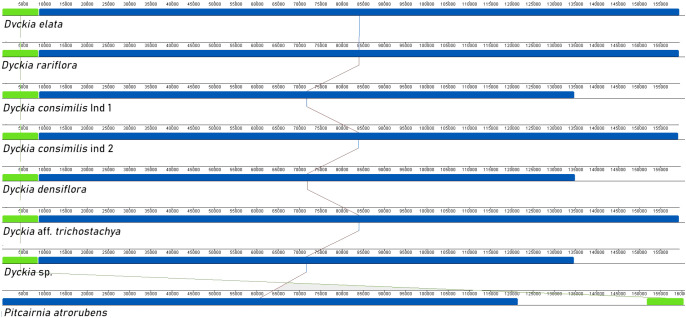
Synteny between *Dyckia* species using Mauve multiple-genome alignment program. Eight different chloroplast genomes are displayed. Colored bars indicate putative syntenic blocks, and connecting lines indicate corresponding blocks.

In accordance with the alignment produced using the chloroplast genome of *Pitcairnia atrorubens* as a reference, all *Dyckia* species exhibited the same number and orientation of syntenic blocks with no detected inversions (**Figure 2**). However, the only noTable difference was a reorganization in the small syntenic block (highlighted in green) that shifted from the beginning of the alignment in *Dyckia* to the end in *Pitcairnia atrorubens*, due to differences in the determination of the LSC region. The noTable decrease in the extent of the second syntenic block (highlighted in blue) in *D. consimilis*, *D. densiflora*, and *D* sp. between 13,200 pb and 16,000 pb overlaps with the IRA region in the plastomes here sequenced.

**Figure 3:**
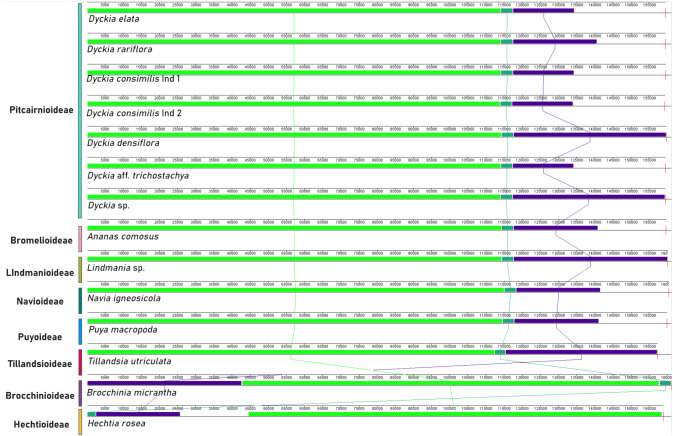
Synteny between Bromeliaceae sub-families using Mauve multiple-genome alignment program. 14 different chloroplast genomes are displayed among all eight Bromeliaceae sub-families. Pitcairnioideae is represented by seven specimens of *Dyckia* genus; Bromelioideae is represented by *Ananas comosus*; Lindmanioideae by *Lidmania*. sp; Navioideae by *Navia igneosicola*; Puyoideae by *Puya macropoda*; Tillandsioideae by *Tilandsia utriculate*; Brocchinioideae by *Brocchinia micrantha* and Hechtioideae by *Hetchia rosea*. All cloroplastidial genomes were available in GenBank. Colored bars indicate putative syntenic blocks, and connecting lines indicate corresponding blocks.

On the other hand, the alignment of all *Dyckia* samples and seven other species representing all subfamilies in Bromeliaceae reveals a high level of synteny in the family with three distinct conserved blocks (**Figure 3**). In this analysis, the small syntenic block (highlighted in dark green) overlaps with the border of the IRB and SSC region. Similar to what was observed in the first alignment, the second syntenic block (highlighted in blue) experiences a decrease in extent over the SSC and IRA border, resulting in unaligned regions in some cases, particularly in the IRA region (between 135,000 bp and the end of the alignment). The rearrangement of the syntenic block order in *Brocchinia micrantha* and *Hechtia rosea* may result from plastome assembly artifacts in NCBI or procedural errors in genome annotation of LSC and SSC region boundaries in the analyzed individuals.

**Figure 4:**
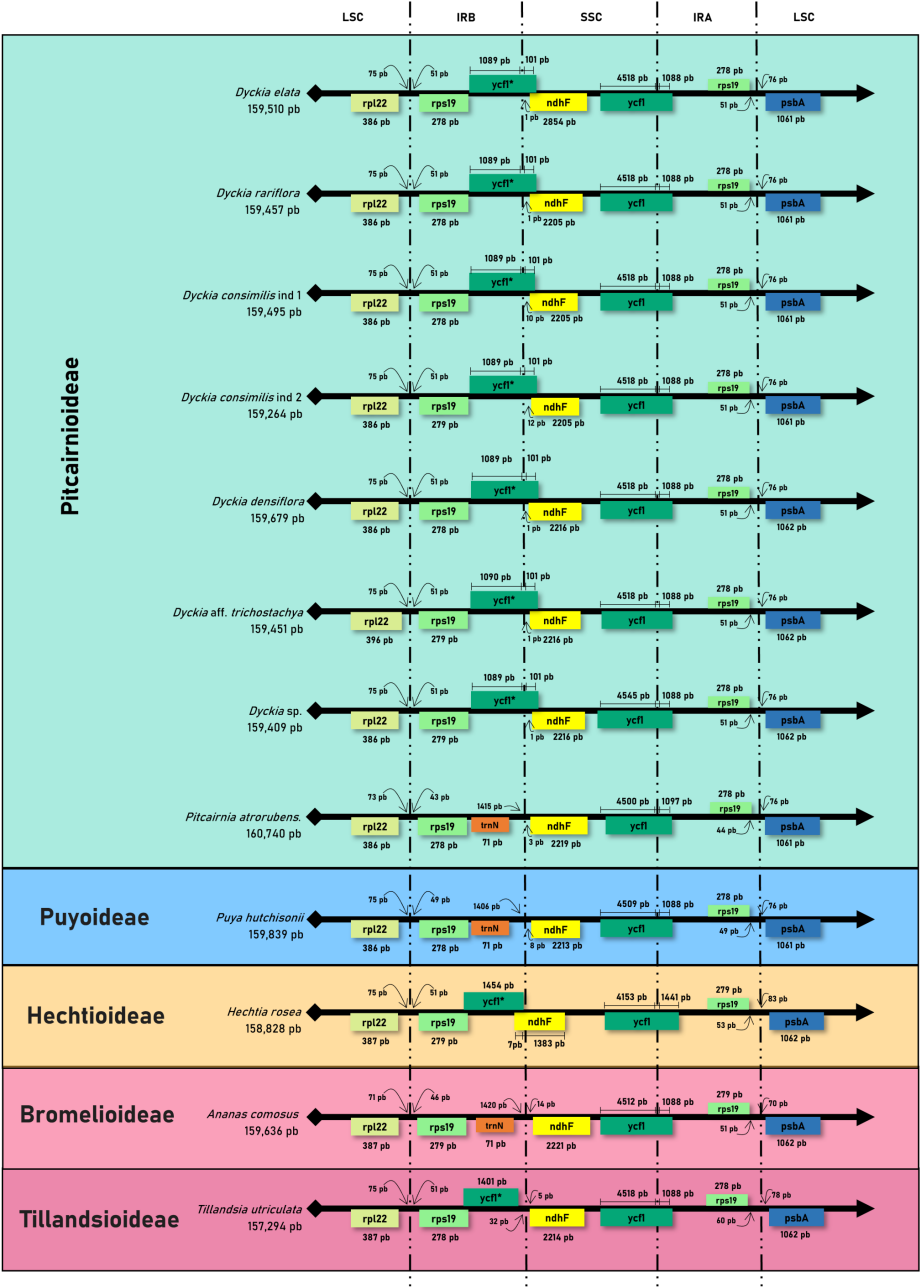
Comparative analysis of the border positions of LSC, SSC, and IR regions across chloroplastial genome sequences from 12 taxa belonging to the Bromeliaceae family.

The analysis of the expansion and contraction of the boundaries in the IRs regions showed that although there is a high conservation of the type, size, and orientation of genes present in the boundaries of LSC and SSC among *Dyckia*, the absence of the *ycf1* fragment in *Puya hutchisonii* was noteworthy (**Figure 4**). The transition of the IRB and SSC regions was characterized by the presence of the *ycf1* fragment, whose length and distance from the SSC border varied considerably. In *Dyckia* species and *Pitcairnia atrorubens*, the *ycf1* fragment extends by 101 and 71 bp into the SSC region, respectively. In *Tillandsia utriculata*, although of similar length, the *ycf1* fragment ends 32 bp before the boundary. Furthermore, within *Dyckia* species, variations (ranging from 1 to 12 base pairs) were identified in the length between the *ndhF* gene and the IRB-SSC region boundary. However, in *Dyckia elata*, a significant expansion of the gene by 649 bp was detected compared to other species.

### 3.3 Detection of Sequence Repeats and Polymorphisms in *Dyckia*

**Figure 5:**
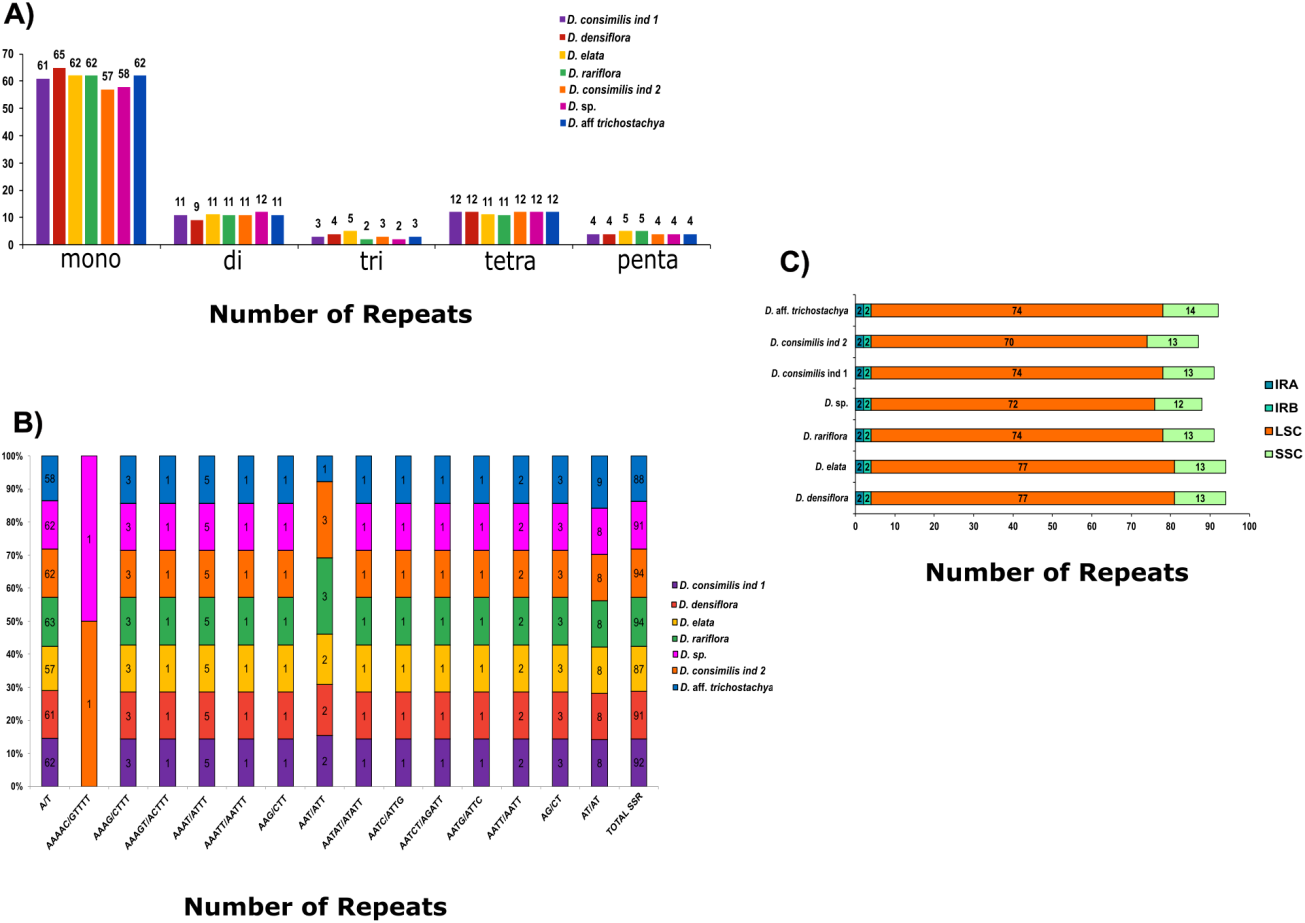
Classification, motifs and chloroplast region occurrence of single sequence repeats (SSR) in the chloroplast genomes of *Dyckia*. **(A)** Number of SSR types (mono-, di-, tri-, tetra-, penta-, and hexanucleotides) present in the seven chloroplast genomes; **(B)** Number of different SSR motifs in the plastomes; **(C)** Number of SSR in the different chloroplast genome regions among species.

We identified 637 simple sequence repeats (SSR) among *Dyckia* species. The species with the most SSR were *D. elata* and *D. densiflora* (94) followed by *D.* aff*. trichostachya, D. rariflora*, and *D. consimilis* ind 1 (92, 91, and 91 respectively). The species with less SSRs found were *D. consimilis* ind 2 (87) and *Dyckia* sp (88). The most common type of SSRs motifs found was mononucleotide (67.03%), followed by tetra and dinucleotides with 11.93% and 12.87%, respectively (**Figure 5A**). Although the SSR motif recurs in all *Dyckia* species, two species have diverged from this pattern: AAAAC/GTTTT was found only in *D. elata* and *D. rariflora*, and the motif AAT/ATT is present in all species except *D. rariflora* (**Figure 5B**). Among *Dyckia* the SSR were more abundant in LSC (81.32%) and less in the IR regions (4.40%) (**Figure 5C**).

**Figure 6:**
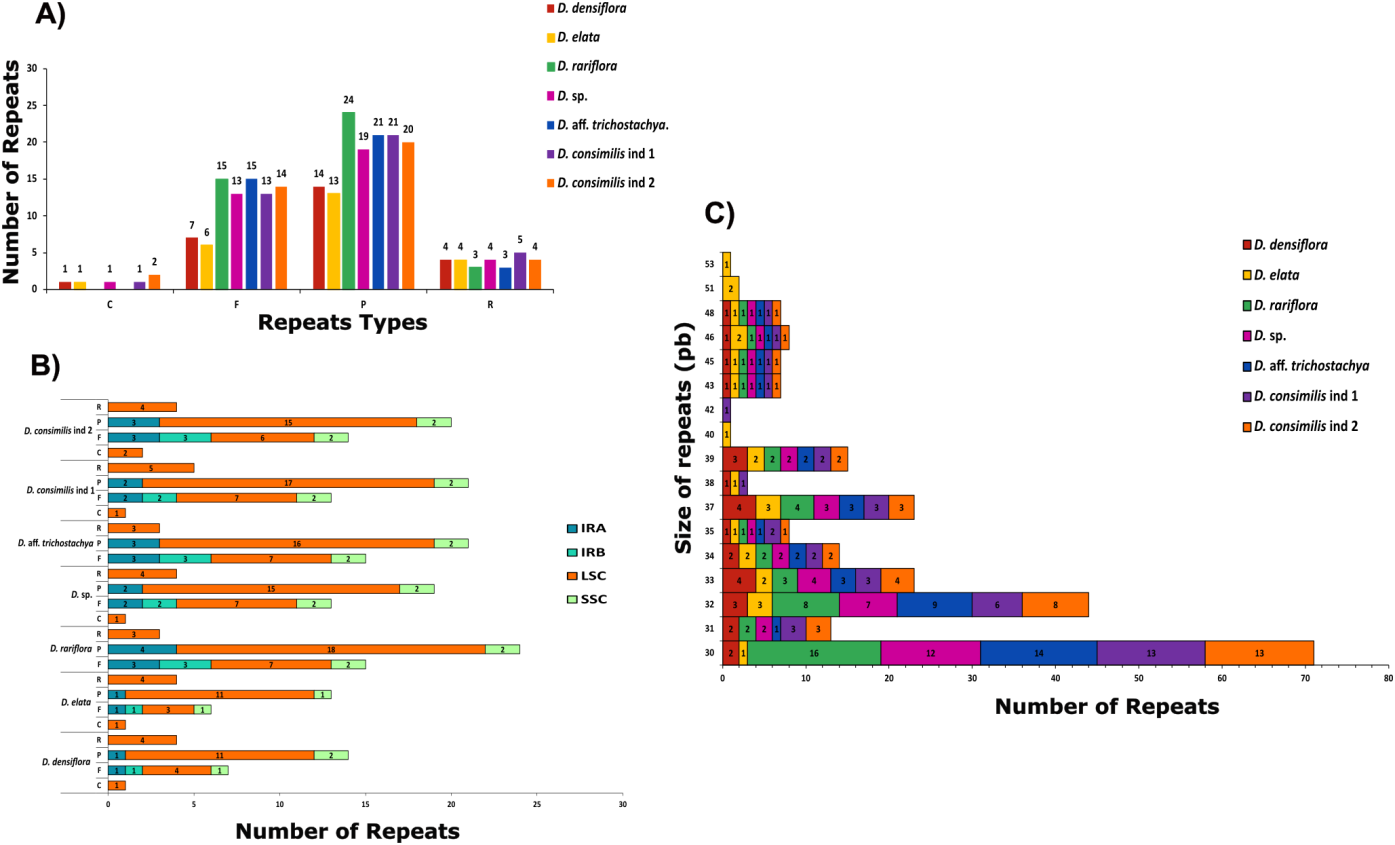
Classification, chloroplast region occurrence and size quantification of dispersed repeats in the chloroplast genomes of *Dyckia*. **(A)** Distribution of different types of repeats: F=Forward, P=Palindrome, R=Reverse and C=Complement; **(B)** Number of repeats in the different chloroplast genome; **(C)** Size quantification of repeats between *Dyckia*.

The quantification of dispersed repeats varied considerably among the species. We observed 42 in *D. rariflora*, 40 in *D. consimilis*, 39 in *D.* aff *trichostachya*, 37 in *D.* sp, 26 in *D. densiflora*, and only 24 in *D. elata.* The most prevalent type in *Dyckia* were palindromic totaling 208 instances. Nevertheless, we also found 82 forward repeats, 27 reverse, and just 6 complement repeats (**Figure 6A**). Similarly to the distribution of SSRs, the majority were found in the LSC (71%), with the least in the IRB (6.5%). Reverse and complement types were only found in the LSC, and although it had the highest number of palindromic repetitions (Vera-Paz et al., 2022), it was not possible to find a complement type in *D. rariflora* (**Figure 6B**). Despite having the lowest quantity of dispersed repeats, *D. elata* exhibited the longest repeats (54 and 53 bp), and it was also the only species to present repeats of 40 bp (**Figure 6C**).

**Figure 7:**
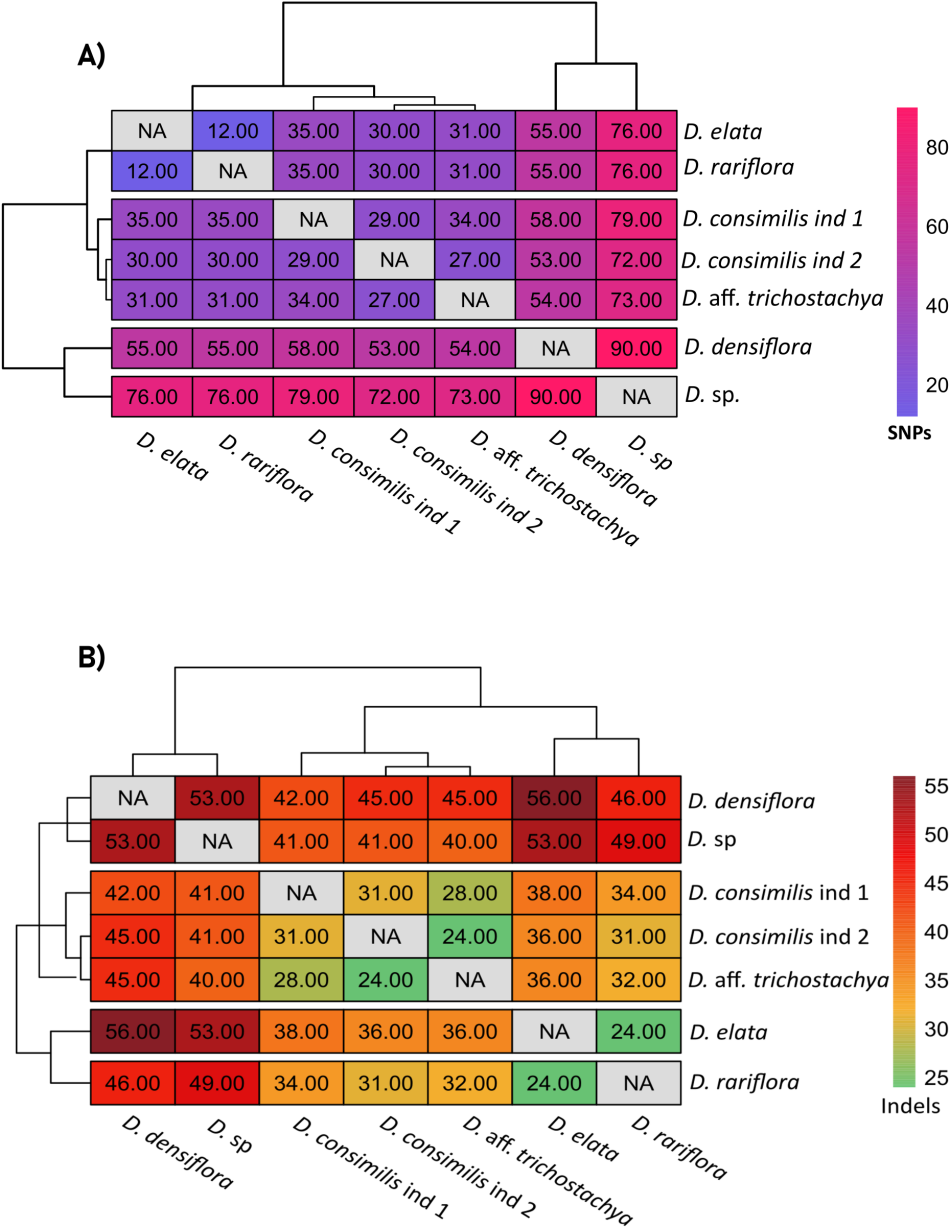
Heatmaps representing the pairwise comparison of SNPS and indels among *Dyckia* spp. **(A)** Heatmap showing the pairwise comparison of single nucleotide polymorphisms (SNPs) among *Dyckia* species. **(B)** Heatmap showing the pairwise comparison of insertions and deletions (indels) among *Dyckia* species.

1035 SNPs were identified across the samples, with the majority (71%) occurring in the single-copy regions LSC and SSC (71% and 22% respectively). The most frequently observed nucleotide substitution was A/T (128 SNPs), and C/G substitutions (26 SNPs) were the rarest. In a pairwise comparison, the most significant divergence in SNPs quantification occurred between *D. densiflora* and *D.* sp. (90 SNPs), which could not be grouped into any similarity block among the evaluated specimens. The lowest number of SNPs was detected between *D. rariflora and D. elata* (12 SNPs), with all substitutions found in the LSC and SSC regions. The intermediate differences in SNP quantification allowed the formation of a group comprising the two individuals of *D. consimilis* (29 SNPs between them) and *D.* aff. *trichostachya* (**Figure 7A**).

Although less frequent than SNPs, indels were also detected as a means of assessing genetic diversity among the plastomes. A total of 823 indels were found among the chloroplast genomes of *Dyckia*, occurring exclusively in the single-copy regions LSC and SSC (**Supplementary Table S3**) The lowest divergence in indel quantification was observed between the species *D. elata* and *D. rariflora*, and *D. consimilis* ind 2 and *D.* aff. *trichostachya* (24 indels). The highest divergence was found between *D. densiflora* and *D. elata* (Darshetkar et al., 2019), as well as between *D.* sp and *D. densiflora* (53 indels) (**Figure 7B**).

**Figure 8:**
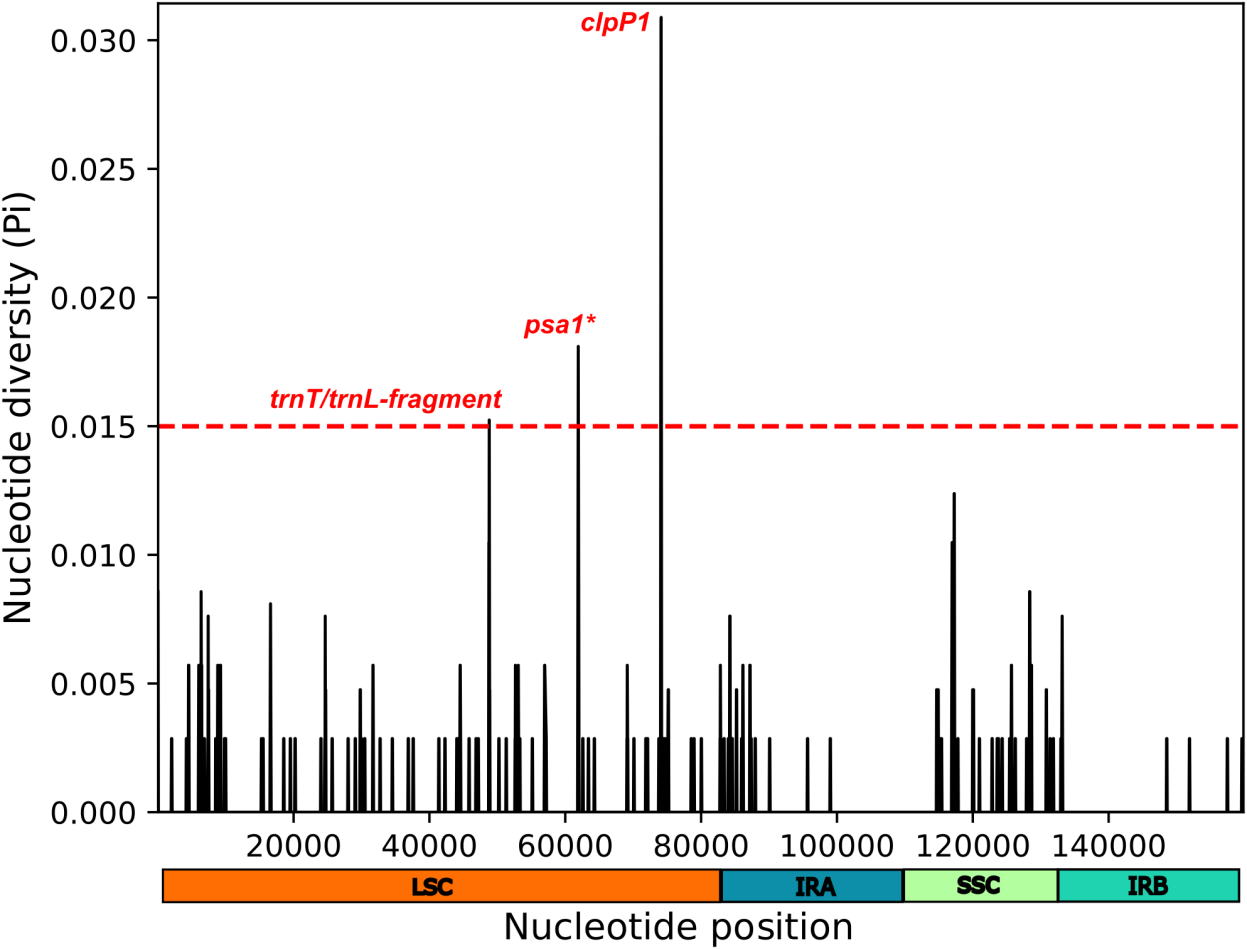
Sliding window analysis conducted on the alignment of chloroplast genomes from *Dyckia* spp. Highlighted is region with high nucleotide variability (Pi > 0.015). Pi denotes the nucleotide diversity within each window, with a window length of 200 bp and step size of 50 bp. The symbol * indicates a gene fragment.

Considering pi > 0.015, the sliding window analysis was able to find three regions of high polymorphic diversity among *Dyckia* all located in LSC region (**Supplementary Table S6**) The highly conserved *clpP1* gene, associated with protein denaturation and cellular development maintenance, *psa1* gene involved in the synthesis of GDP-mannose and the intergenic region *trnT* and *trnL* a non-coding region commonly used as a barcode for inferring relationships between several botanical lineages. All these regions, typically not considered traditional chloroplast markers in phylogenetic analyses or barcoding, may serve as potential hotspots of diversity and important molecular markers for *Dyckia* (**Figure 8**).

### 3.4 Phylogenomic analysis

**Figure 9:**
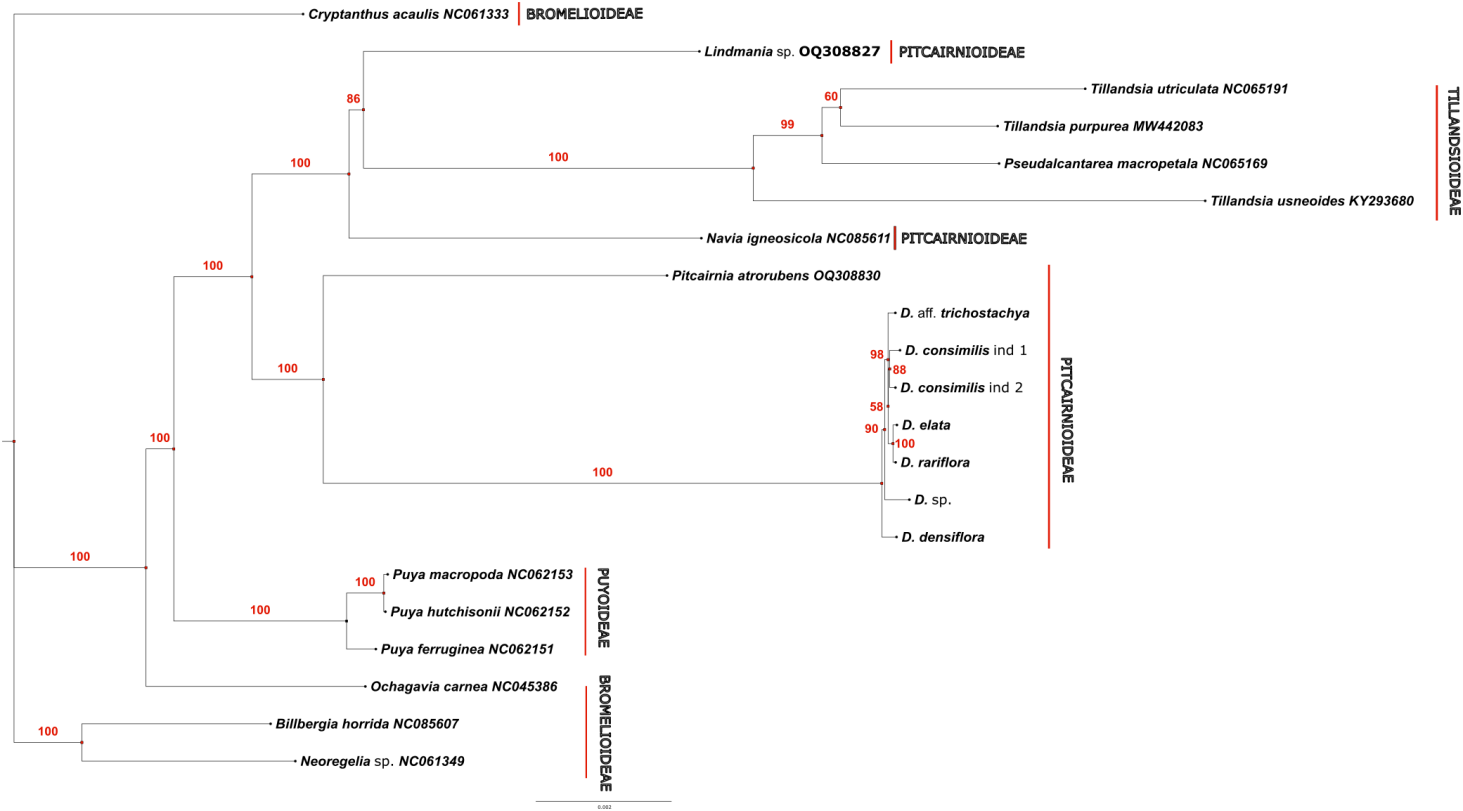
Phylogenetic tree performed under Maximum Likelihood for 21 complete chloroplast genomes of Bromeliaceae species.

The ModelFinder software found the best score for the model TVM+F+R2 for the whole partition of characters. The phylogenetic tree (rooted on *Lindmania*) separated two main groups of bromeliads, the first with Tillandsioideae taxa (*Tillandsia* and *Pseudoalcantarea*, 100% Bootstrap Support-BS) and the remaining with taxa of subfamilies Bromelioideae, Puyoideae, Navioideae and Pitcairnioideae, which were closer to *Dyckia* (**Figure 9**). Within this clade, *Navia* (Navioideae) was sister (84% BS) to the remaining taxa in Bromelioideae, Puyoideae and Pitcarinioideae. This clade was in turn divided in two subclades (each with 100% BS). One subclade had species of *Puya* (Puyoideae) as sister to all taxa in Bromelioideae (*Ochagavia, Cryptanthus, Billbergia* and *Neoregelia*, 100% BS). The other subclade presented *Pitcairnia atrorubens* as sister (100% BS) to all species of *Dyckia*. The monophyly of *Dyckia* was well supported (100% BS). Within *Dyckia*, branch lengths were much shorter, but *D. densiflora* was sister to remaining species with 90% BS. The next level corresponds to a branch with very low support (58% BS) which corresponds to a methodological polytomy, with *Dyckia* sp. as sister to two smaller groups. One corresponds to *D. rariflora* as sister to *D. elata* (100% BS) and the other the two samples of *D. consimilis* together (88% BS) and sister (98% BS) to *D.* aff. *trichostachya*.

## 4 Discussion

As shown in **Figure 1** and **Table 1**, the complete chloroplast genome sequencing of related *Dyckia* species revealed a high degree of structural and gene composition conservation. However, we detected minor variations in gene order, consistent with previous observations in other Bromeliaceae species (Redwan et al., 2015; Liu et al., 2022). This pattern has also been widely reported in species with limited distribution and recent evolutionary radiation (Niu et al., 2017; Li et al., 2020; Xu and Xu, 2021). Although similar in length, small variations due to the expansion and contraction of the IR/SSC regions were observed among individuals of the same species (**Table 1**). For example, in *Dyckia consimilis*, the length of the LSC region varied from 87,331 bp to 87,104 bp. Despite this, unlike other Bromeliaceae genera such as *Hechtia* and *Vriesea*, no inversions were found in the plastomes of *Dyckia* species (Vera-Paz et al., 2022; Ramírez-Morillo et al., 2023).

Even though its function is not yet fully understood, the open reading frame 1 (*ycf1*) fragment appears to be a noTable synapomorphy of the chloroplast genome in Bromeliaceae. Its unique positioning, extending between the SSC border and the repetitive regions of the plastome, along with its apparent essential role in plant viability, underscores its potential as a promising barcode marker for terrestrial plants (Drescher et al., 2000; Neubig et al., 2009). Given the significant potential for recombination and homologous rearrangement, along with the high substitution rates in inverted regions that affect structural stability, the borders between the LSC, IR, and SSC regions are common sites of structural variation and mutations (Kim and Lee, 2005; Yue et al., 2008; Xiong et al., 2009; Zhu et al., 2016). While all evaluated *Dyckia* species displayed a truncated *ycf1* fragment at the IRB/LSC border, variations in the length of the *ndhF* gene, as observed in *D. elata*, along with small modifications in the distance between plastid region borders and the gene, support its potential use as a molecular marker in this genus. The overlap between the *ycf1* and *ndhF* genes, along with their structural variations, has been widely linked to the expansion of the IR region (Dong et al., 2015). This suggests rapid positive selection acting on these genes, with their rate of structural variation potentially providing more insightful information for the development of DNA barcodes than traditionally used genes like *rbcL* or *matK* (Amar, 2020).

In our screening for potential DNA barcodes, we identified three sequences—*clpP1*, *psaI*, and the *trnT-trnL* intergenic region—with higher molecular variability that were not included in the most recent phylogenetic analysis proposed for *Dyckia* (Gomes-Da-Silva et al., 2019). Despite their promising variability, the informative potential of these regions remains underexplored in previous studies (Krapp et al., 2014). The diversity found in barcodes that are not conventionally used in molecular analyses for *Dyckia* and other bromeliad lineages highlights the need for taxon-specific barcodes or the use of ‘super-barcodes’ with complete plastome sequences to enhance interspecific resolution in these taxa.

Repetitive sequences play an important role in the structural rearrangement of the chloroplast genome in various plant species (Wheeler et al., 2014). Similarly, cpSSRs have been widely used as molecular tools in population genetics studies, species delimitation, and analyses of hybridization and introgression in related species, largely due to their co-dominant expression and polymorphism (Gonçalves-Oliveira et al., 2020; Aecyo et al., 2021; Pinheiro et al., 2021).

The repetitive content of *Dyckia* chloroplasts, including cpSSRs (94–88) and dispersed repeats (42–24), was higher than that found in *Puya* spp. and other species of the order Poales, such as *Eriocaulon decemflorum*, but still considerably lower than that found in other bromeliads such as *Ananas comosus* and *Tillandsia usneoides* (Redwan et al., 2015; Poczai and Hyvönen, 2017; Darshetkar et al., 2019; Liu et al., 2022). Dispersed repeats are broadly associated with mutational hotspots, chromosomal rearrangements, and localized expansions of chloroplast genome size. Their presence and variability among *Dyckia* species suggest considerable evolutionary plasticity and highlight their potential as informative polymorphic markers at the population level. These features, as well as cpSSR motifs, may be particularly valuable for distinguishing closely related species and for informing the design of effective *in situ* conservation strategies (Ebert and Peakall, 2009).

SNPs and indels are the most common types of mutations in cpGenomes. The low SNPs diversity between *D. elata* and *D rariflora* may support the previously suggested hypothesis of synonymization between the species (Guarçoni, 2014). However, considering their restricted distribution and high morphological similarity, analyses using other molecular markers with higher mutation rates, capable of elucidating recent evolutionary events, are needed to further clarify the similarity between the species. Furthermore, the diversity of indels and SNPs among individuals of the same species, as seen in *Dyckia consimilis*, underscores a possible intra-populational diversity greater than what is typically found in other *Dyckia* spp, highlighting the need for further population studies in *Dyckia* (Hmeljevski et al., 2011; Hirsch et al., 2020).

The phylogeny obtained despite the limited sample of plastomes included, clearly replicates the overall phylogenetic structure of Bromeliaceae of Givnish and colleagues (Givnish et al., 2014). Based on that study, we selected *Lindmania* as an outgroup, and the first group to diverge was Tillandsoideae, followed by Navioideae, since no plastome of Hechtioideae was included in our matrix. Finally, the next part included Puyoideae+Bromelioideae as sister to Pitcairnioideae, and *Pircairnia* as sister to the samples of *Dyckia*, which is altogether a perfect reduced mirror of the phylogenetic structure of Givnish and colleagues. *Pitcairnia* as sister of *Dyckia* is also perfectly congruent with other studies in Pitcarinioideae (Pinangé et al., 2017; Gomes-Da-Silva et al., 2019; Moura et al., 2019), since *Fosterella* and *Deuterocohnia* are not sampled in our study. All of the relationships above attained high support (86-100% BS), except the relationship between the two taxa in *Tillandsia*, which are probably closely related and with low divergence.

Although the *Dyckia* samples analyzed in this study have not previously been evaluated collectively in other phylogenetic analyses, with the exception of *D. rariflora* and *D. densiflora*, which were previously studied together (Pinangé et al., 2017), their restricted distribution in the southern portion of the Espinhaço Range supports the formation of a monophyletic clade of *Dyckia*, composed of microendemic species from this region. The polytomy observed between *Dyckia* sp. and the remaining species may be attributed to their geographical distribution along the southern Espinhaço Mountain Range: *D. elata* and *D. rariflora* exhibit remnant populations in the Serra do Caraça, about 100 km away from the other species analyzed, which occur throughout the “Sinclinal Moeda” mountains in small, isolated populations of very few individuals, separated by civil infrastructure, roads, and mining areas.

The low molecular variability observed among *Dyckia* species, even using non-coding regions, may reflect the genus’s recent history of radiative evolution, particularly in its center of origin, as well as the influence of evolutionary strategies such as self-fertilization and introgression, which are common within the genus (Hmeljevski et al., 2011; Hirsch et al., 2020; Pinangé et al., 2020). Our findings highlight the increasing need for genomic studies utilizing nuclear and chloroplast markers, aimed at examining a broader range of samples and elucidating the evolutionary dynamics of *Dyckia* at the population level, taking into account geographic and reproductive barriers among threatened microendemic species.

## Data Availability

The data presented in this study are publicly available in the NCBI BioProject repository under accession number PRJNA1273098.
